# Genome sequencing of a metallo-beta-lactamase-producing *Escherichia coli* Ne7 strain isolated from a patient with urinary tract infection in Ahvaz-Iran

**DOI:** 10.1128/mra.01272-24

**Published:** 2025-02-18

**Authors:** Ihab Rasmi Hassan, Seyedeh Elham Rezatofighi, Hossein Motamedi, Md. Tanvir Rahman

**Affiliations:** 1 Department of Biology, Faculty of Science, Shahid Chamran University of Ahvaz, Ahvaz, Iran; 2 Department of Microbiology and Hygiene, Faculty of Veterinary Science, Bangladesh Agricultural University, Mymensingh, Bangladesh; University of Maryland School of Medicine, Baltimore, Maryland, USA

**Keywords:** uropathogenic *Escherichia coli*, metallo-beta-lactamase, extensively drug-resistant, urinary tract infection

## Abstract

We report the draft genome sequence of an extensively drug-resistant and metallo-beta-lactamase-producing *Escherichia coli* Ne7 strain isolated from a urinary tract infection patient. Under the O8:H9 serotype and F phylogenetic group, this strain had a sequence type of ST648, five replicon plasmids, 16 virulence genes, and 21 antibiotic resistance genes.

## ANNOUNCEMENT

Urinary tract infection (UTI) is one of the most common bacterial infectious diseases, mainly caused by uropathogenic *Escherichia coli* (UPEC) (1). Antibiotic-resistant *E. coli* strains pose a significant global health threat, particularly in clinical settings.

In 2022, we isolated *E. coli* Ne7 from the urine of a patient with UTI in Ahvaz-Iran by culture on MacConkey agar and blood agar. After overnight incubation at 37°C, single colonies were picked up for identification by the biochemical and microbiological tests including oxidase, SIM, TSI, MR-VP, and Simmons' citrate (2). The antimicrobial resistance (AMR) profile was evaluated using the Kirby-Bauer disc diffusion method according to Clinical and Laboratory Standards Institute (CLSI) 2024 (3). The Minimum Inhibitory Concentration (MIC) of the strain against colistin was done by the broth dilution method (3). The results showed that this strain was resistant to gentamycin, nitrofurantoin, meropenem, imipenem, ceftriaxone, kanamycin, trimethoprim-sulfamethoxazole, nalidixic acid, cefoxitin, cefepime, ampicillin-sulbactam, amikacin, tobramycin, cefazolin, fosfomycin, tetracycline, ciprofloxacin, ampicillin, piperacillin-tazobactam, chloramphenicol, and aztreonam but susceptible to colistin. Based on the extensively drug-resistant (XDR) definition (non-susceptibility to at least one drug in all but one or two antimicrobial classes), this strain was considered XDR (4). The metallo-beta-lactamase (MBL)-producing capability was evaluated by the modified carbapenem inactivation method (mCIM) and EDTA-Carbapenem Inactivation Method (EDTA-CIM) (eCIM) tests, and *bla*_NDM_ was detected by PCR (5).

One colony of *E. coli* Ne7 was cultured in the nutrient broth and incubated overnight at 37°C. After centrifugation, the bacterial sediment was used for DNA extraction by Top Bacterial DNA extraction kit (TopazGene; Iran). Whole-genome sequencing was performed using the NovaSeq 6000 sequencer (Illumina). TruSeq DNA sample preparation kit (Illumina) was used to prepare the libraries with 2 × 150 bp paired-end. Trimmomatic (v0.39) was used for quality trimming and adapter clipping of the (6,012,766) raw reads (6). Survived reads (5,990,156) were assembled *de novo* using SPAdes (v3.15.5) (7). Genome annotation was performed by NCBI Prokaryotic Genome Annotation Pipeline (v6.6) (8). AMR genes were predicted by ResFinder (v.4.6.0) (9, 10) and CARD (v.6.0.3) (11). Sequence type, pathogenicity index, inc plasmids, acquired virulence factor genes, serotype, and metabolic functional features were identified by MLST (v.2.0) (12), PathogenFinder (v.1.1) (13), PlasmidFinder (v.2.1) (14), VirulenceFinder (v.2.0) (15), SeroTypeFinder (v.2.0) (16), and RAST (v.2.0) (17), respectively. Default parameters were used for all software.

The total length of the *E. coli* Ne7 genome was 5,281,053 bp with 204 contig. In RAST, the assembled genome showed 397 subsystems with 31% coverage and 1,587 genes. The general characteristics are indicated in Table 1.

**TABLE 1:** Summary of general characteristics of *E. coli* Ne7

Attributes	Values
Total genes	5,357
Coding genes	5,084
Contig N50	113,717 bp
G + C content	50.56%
Serotype	O8:H9
Genome coverage	170×
RNA genes	73
tRNA genes	65
rRNA	3
Non-coding RNA	5
CRISPR arrays	2
Replicon plasmids	IncFIA, IncFIB, IncI (Gamma), IncC, IncFII
Predicted antibiotic resistance genes (ARGs) by ResFinder	21
Predicted ARGs by CARD	76
Predicted virulence genes	16
Pathogenicity index	0.992
Sequence type	ST648
BioSample accession no.	SAMN41098724
BioProject accession no.	PRJNA1105233
GenBank accession no.	JBJIES000000000
Sequence Read Archive no.	SRX26011531
